# Multiplexed P21/MCM-2 Detection Predicts Relapse and May Identify Tyrosine Kinase Inhibitor–Resistant Patients in Clear Cell Renal Cell Carcinoma

**DOI:** 10.1158/2767-9764.CRC-25-0805

**Published:** 2026-05-07

**Authors:** Hazem Abdullah, In Hwa Um, Grant D. Stewart, Chang Wook Jeong, Cheol Kwak, Kyung Chul Moon, Alexander Laird, Elena Frangou, Tim Eisen, Angela Meade, David J. Harrison

**Affiliations:** 1 School of Medicine, University of St Andrews, St Andrews, United Kingdom.; 2 Department of Surgery, University of Cambridge, Cambridge, United Kingdom.; 3 Department of Urology, Seoul National University College of Medicine, Seoul, Republic of Korea.; 4 Department of Pathology, Seoul National University College of Medicine, Seoul, Republic of Korea.; 5 Department of Urology, Western General Hospital, Edinburgh, United Kingdom.; 6 Medical Research Council Clinical Trials Unit at University College London (UCL), Institute of Clinical Trials and Methodology, London, United Kingdom.; 7 Department of Oncology, University of Cambridge, Cambridge, United Kingdom.

## Abstract

Current clinical tools for predicting relapse and guiding adjuvant therapy in clear cell renal cell carcinoma (ccRCC) lack precision, especially in intermediate-risk disease. This study evaluated whether a nonproliferative tumor cell phenotype, P21 (*CDKN1A* inhibitor)–positive and MCM2 (DNA replication protein)–negative (P21^+^/MCM2^−^), could serve as a robust biomarker to improve prognostic stratification and guide postnephrectomy treatment decisions. We used multiplex immunofluorescence and Artificial Intelligence–based image analysis on nephrectomy specimens from three independent ccRCC cohorts: UK arm of the SORCE trial (*n* = 382), Korean (*n* = 71), and Scottish (*n* = 88). An optimal 2% cutoff for P21^+^/MCM2^−^ cells was determined using X-tile software. Additional analyses assessed endoglin/CD105 coexpression, paired primary–metastatic samples (*n* = 41), and associations with adjuvant sorafenib therapy. In two intermediate-risk ccRCC cohorts (SORCE, *n* = 63; Korean, *n* = 71), patients with >2% P21^+^/MCM2^−^ cells had significantly longer time to relapse [HR = 0.17; 95% confidence interval (CI), 0.06–0.54; HR = 0.27; 95% CI, 0.10–0.72]. Prognostic value was confirmed in high-risk (SORCE, HR = 0.43; 95% CI, 0.19–0.99) and all-risk (Scottish, HR = 0.37; 95% CI, 0.14–0.98) cohorts. Notably, patients with high P21^+^/MCM2^−^ levels on placebo fared better than those receiving adjuvant TKI therapy (HR = 0.29; 95% CI, 0.16–0.50). In 41 paired samples, 85% showed higher P21^+^/MCM2^−^ abundance in metastases than in primary tumors. As a conclusion, P21^+^/MCM2^−^ cell count is a robust biomarker that refines relapse risk stratification in ccRCC and identifies patients who may not benefit from adjuvant tyrosine kinase inhibitor (TKI) therapy. High levels of these nonproliferative, senescent-like cells suggest tumor dormancy and a more favorable outcome without treatment.

**Significance:**

After surgery for clear cell renal cell carcinoma, relapse risk and benefit from adjuvant therapy remain uncertain. We identify a distinct P21^+^/MCM2^−^ tumor phenotype, nonproliferative and senescent-like, linked to favorable outcomes. Across three cohorts, >2% of these cells reduced relapse risk, but benefit was lost with adjuvant TKIs.

## Introduction

Renal cell carcinoma (RCC) is the most common kidney cancer in adults, accounting for 90% to 95% of all malignancies of the kidney (1). Kidney cancer accounts for about 2% of all cancer diagnoses and deaths globally, with incidence rates usually greater in developed countries (2). The majority of kidney cancer fatalities are caused by the most widespread subtype, clear cell RCC (ccRCC; ref. 3).

Given the critical role of angiogenesis in ccRCC, markers such as CD105 have been used to identify endothelial cells within RCC (4). Angiogenesis in this context is largely driven by *VHL* loss and subsequent HIF-mediated VEGF overexpression, which led to the development of VEGFR-targeted tyrosine kinase inhibitors (TKI) as systemic therapies (5). In the mid-2000s, drugs like sorafenib, sunitinib, and pazopanib transformed the treatment of metastatic RCC, leading to research into their potential use as adjuvant therapies after surgery (6). The SORCE trial, a large placebo-controlled study of sorafenib in intermediate/high-risk RCC that involved 1,711 patients, found that adjuvant sorafenib did not improve disease-free survival for up to 3 years of treatment (7). Whereas their adjuvant role remains under debate, later-generation TKIs, including axitinib and lenvatinib, continue to represent standard-of-care options in the metastatic setting (8).

At the molecular level, disruption of the cell cycle is a key component of many malignancies, including RCC (5). Cell-cycle status is commonly assessed by evaluating cellular morphology, such as increased cell size as well as through molecular markers, including P21 and P16, which indicate cell-cycle arrest; MCM2, which marks active proliferation; and Lamin B1, whose loss is associated with cell-cycle exit and nuclear remodeling (9). P21 (*CDKN1A*) is a cyclin-dependent kinase inhibitor that can cause cell-cycle arrest or senescence and enforces the G_1_/S checkpoint in response to stress usually through p53 (10). Prior studies demonstrated a correlation between p21 expression and ccRCC outcomes, as patients with localized ccRCC who had elevated nuclear P21 had higher survival rates; however, the opposite was true for patients with metastatic ccRCC (11). P16 (*CDKN2A*) functions as a tumor-suppressor by inhibiting CDK4/6, reinforcing G_1_ arrest (12). Also, Lamin B1, a key structural protein of the nuclear lamina, is often downregulated in arrested or senescent cells, reflecting changes in nuclear architecture associated with cell-cycle exit (13). MCM2 is a licensing factor for DNA replication that is necessary for S-phase proliferation. Cells express MCM2 during all phases of the cell cycle except G_0_ (as well as other members of the MCM2–7 complex; ref. 14). Higher tumor grade, advanced stage, and a poorer prognosis have all been associated with elevated MCM2 expression in malignancies (15). *In situ* investigations have demonstrated that MCM2 indices significantly outperform Ki-67 indices in ccRCC (median ∼42% vs. 7% in one series), indicating a higher percentage of cancer cells with the capacity to multiply (16). All these findings point to a fine balance in tumor biology between cell-cycle arrest and proliferative drive. Composite biomarkers that have predictive value beyond single indicators alone may be produced when proliferation and arrest signals coexist in cancer cells. This establishes the foundation for investigating dual-phenotype cells, such as P21-positive cells/MCM2-negative (P21^+^/MCM2^−^), which could be a quiescent or senescent fraction within tumors.

Robust biomarkers are much needed to guide postnephrectomy decision-making due to the shortcomings of current clinicopathologic risk models and risk/benefit of adjuvant therapies (17). Through the use of tumor stage, grade, and other variables, clinical prognostic models [such as the UISS, SSIGN, and Leibovich score (LS)] categorize patients into low-, middle-, and high-risk groups for relapse (18). Despite this, it is still difficult to predict relapse with any degree of accuracy, particularly for localized ccRCC that is intermediate- or high-risk. A proportion of intermediate-risk cancers exhibit aggressive behavior, whereas other purportedly high-risk patients never experience a relapse (19).

The tumor microenvironment in ccRCC consists of a dynamic mix of proliferating, quiescent, and arrested cells (20). We hypothesize that tumor behavior in ccRCC may be influenced by a distinct population of quiescent cells, defined by a P21^+^/MCM2^−^ phenotype. This hypothesis stems from histologic observations of large, morphologically distinct cells on hematoxylin and eosin staining which may represent a quiescent or senescent subpopulation. We propose that the abundance of these noncycling cells could serve as a biomarker for relapse risk and suggest a subgroup at risk of harm from adjuvant TKIs in ccRCC.

## Materials and Methods

### Study population

This study incorporated three independent cohorts of patients with ccRCC (Table 1). The first cohort (*n* = 382) was drawn from UK-based patients enrolled in the SORCE trial (ClinicalTrials.gov identifier: NCT00492258), a multicenter, randomized, double-blind phase III study designed to compare 1 and 3 years of adjuvant sorafenib versus placebo following nephrectomy (7). Patients within the SORCE cohort were stratified according to the LS into intermediate- (LS3, LS4, and LS5) and high-risk (LS6 and higher) subgroups (21). The intermediate-risk subgroup was used as the training set in this analysis, whereas the high-risk subgroup served as a subsequent test set to assess the applicability of this biomarker beyond the intermediate-risk population.

**Table 1.** Clinicopathologic characteristics of patients with primary disease only, across the SORCE, Korean, and Scottish cohorts.

Cohort	SORCE (*N* = 382)	Korean (*N* = 71)	Scottish (*N* = 88)
Gender	​	​	​
Male, 366 (68%)	266 (70%)	55 (77%)	45 (51%)
Female, 175 (32%)	116 (30%)	16 (23%)	43 (49%)
Age (years)	​	​	​
Median	59	55	65
Range	30–81	30–81	31–90
T stage	​	​	​
1, *n* = 104 (19%)	37 (10%)	25 (35%)	42 (48%)
2, *n* = 118 (22%)	84 (22%)	22 (31%)	12 (14%)
3 or 4, *n* = 319 (59%)	261 (68%)	24 (34%)	34 (39%)
ISUP nuclear grade	​	​	​
1, *n* = 61 (11%)	7 (2%)	25 (35%)	29 (33%)
2, *n* = 150 (28%)	92 (24%)	19 (27%)	39 (44%)
3, *n* = 244 (45%)	199 (52%)	26 (37%)	19 (22%)
4, *n* = 86 (16%)	84 (22%)	1 (1%)	1 (1%)
Leibovich risk score	​	​	​
Low (0–2), *n* = 35 (6%)	0	0	35 (40%)
Intermediate (3–5), *n* = 275 (51%)	167 (44%)	71 (100%)	37 (42%)
High (>6), *n* = 231 (43%)	215 (56%)	0	16 (18%)
Relapsed	​	​	​
No, *n* = 334 (62%)	210 (55%)	55 (77%)	69 (78%)
Yes, *n* = 207 (38%)	172 (45%)	16 (23%)	19 (22%)
Treatment	​	​	​
Placebo	110 (29%)	NA	NA
1-year sorafenib	131 (34%)	NA	NA
3-year sorafenib	141 (37%)	NA	NA

Variables include sex distribution, age, tumor stage, nuclear grade, Leibovich risk score, disease relapse, and adjuvant treatment regimen (SORCE only).

The second cohort, serving as a geographically separate validation group (*n* = 71), comprised a longitudinal cohort of patients with LS intermediate-risk ccRCC, treated surgically at Seoul National University Hospital, South Korea. This cohort was collected under institutional ethical approval (IRB number: H-1908-133-1057) from Seoul National University Hospital.

The third cohort (*n* = 88) was a longitudinal collection of patients diagnosed with ccRCC, treated by nephrectomy in Edinburgh, Scotland. Formalin-fixed, paraffin-embedded (FFPE) tumor specimens were obtained from the pathology archive in Edinburgh. All tissue samples were collected prior to the initiation of any systemic therapy. Ethical approval and access to clinical data and biospecimens for this retrospective study were granted by the Bioresource on behalf of NHS Lothian (IRAS reference: 15/ES/0094).

Lastly, tumor samples from 41 patients with ccRCC, each with matched primary and metastatic lesions, were selected from a prospectively maintained clinical database in Edinburgh (paired cohort). These patients had either renal vein or inferior vena cava thrombus and/or distant metastases, identified at the time of nephrectomy or during follow-up (22). Ethical approval for the use of archived material was obtained from the Lothian Regional Ethics Committee (references: 08/S1101/41 and 10/S1402/33).

### Antibody optimization

All antibodies were optimized for brightfield immunoperoxidase and multiplex immunofluorescence (mIF) using tissue microarrays (TMA) comprising 120 samples of a variety of normal and cancerous tissues (RCC, breast, stomach, colon, prostate, tonsil, spleen, etc.). Optimization was performed by testing a range of antibody dilutions and antigen retrieval conditions to determine optimal staining performance. Conditions were selected based on signal intensity, minimal background staining, and preservation of expected cellular localization. Positive and negative control tissues were included to confirm antibody specificity. Following optimization, all analyses in the study cohorts were performed on full-face whole tissue sections to ensure comprehensive assessment of intratumoral heterogeneity.

### Immunohistochemistry

Sections (3 μm) were rehydrated in xylene and then in graded alcohol (100%, 100%, 80%, 50%) and rinsed in water. Heat-induced antigen retrieval was performed in an automatic pressure cooker (5 minutes, 99°C, 0.01 mol/L citrate buffer, pH 6.0). Endogenous peroxidase activity was blocked with 3% hydrogen peroxide (5 minutes), followed by a 5-minute wash in 0.1% TBST. Serum-free block solution (Agilent, X090930-2) was applied for 10 minutes. Primary antibodies P21 (Abcam #ab109520, 1:200), Lamin B1 (Novus #NBP1-42594, 1:1,000), P16 (Roche #06695248001, prediluted), and MCM2 (Cell Signaling #4007, 1:250) were diluted in DAKO diluent. TMA and renal sections were incubated with EnVision horseradish peroxidase (HRP)-conjugated anti-mouse or anti-rabbit secondary antibodies, developed with DAB chromogen (Agilent, K346711-2; 10 minutes), and then mounted in DPX (Sigma-Aldrich).

### mIF

FFPE blocks were sectioned at 3 μm and dried at 65°C. Antigen retrieval was performed in a pressure cooker (5 minutes, pH 6 sodium citrate buffer), followed by washing in TBST (0.1% Tween 20). Endogenous peroxidase and nonspecific signals were blocked with 3% hydrogen peroxide (Sigma #H1009) and serum-free protein block (Agilent X090930-2). MCM2 (Cell Signaling #4007, 1:500) was incubated for 1 hour at room temperature and then with anti-rabbit HRP (Agilent #K400311-2, 30 minutes) and detected with TSA FITC (Akoya #NEL741001KT, 1:50). Bound antibodies were stripped by microwaving in pH 6 sodium citrate buffer for 17 minutes. P21 (Abcam #ab109520, 1:400) and CD105 (HPA067440, 1:600) were visualized with TSA cyanine 3 (Akoya #NEL744001KT, 1:50) and TSA cyanine 5 (Akoya #NEL745001KT, 1:50), respectively. Sections were counterstained with Hoechst 33342 (Thermo Fisher Scientific #H3570, 1:100) and mounted with Prolong Gold anti-fade (Thermo Fisher Scientific #P36930).

### Section scanning

Whole-slide images were captured using the Zeiss Axio Scan Z1 slide scanner using four fluorescence channels, DAPI (461 nm), FITC (519 nm), Cy3 (570 nm), and CY5 (670 nm). Slides from every cohort were scanned with the same profile to maintain consistency.

### BioImage analysis

Fluorescence whole-slide images were analyzed using the Indica HALO platform (v3.4.2986.151) with HALO AI (Indica Labs, https://www.indicalab.com/halo-ai). A custom nuclear segmentation classifier was trained on DAPI signal, with manually annotated nuclei and background regions across multiple cohort samples, to optimize nuclear detection and reduce false segmentation from FFPE autofluorescence. Segmentation accuracy was confirmed by visual inspection. Tumor regions were manually annotated on multiplexed slides using matched H&E-stained sections for reference. Marker quantification within tumor areas was performed with the HighPlex FL module, phenotyping individual cells by nuclear colocalization of MCM2, P21, and CD105 using fluorescence thresholds (FITC, Cy3, and Cy5). Fluorescence intensity thresholds for each marker were established during analysis of the training cohort (SORCE intermediate-risk cohort), using positive control tissues to define signal ranges corresponding to true nuclear staining and to distinguish this from background fluorescence. Individual cells were then classified as marker-positive or -negative based on these predefined thresholds, with P21^+^/MCM2^−^ cells defined by nuclear positivity for P21 and absence of MCM2 signal. The initial assessment of positive staining was performed by eye, with three of the authors (H. Abdullah, I.H. Um, and D.J. Harrison) independently agreeing on the classification criteria.

To ensure reproducibility across staining batches and cohorts, TMA control cores containing known positive tissues were included in each multiplex staining run. These controls were used to monitor staining performance and signal consistency. Where minor batch-to-batch variation in fluorescence intensity occurred, thresholds were adjusted conservatively based on control tissue signal distributions to preserve equivalent biological classification. All thresholding and adjustments were performed independently of clinical outcome data, and the same classification framework was applied to the validation and test cohorts.

### Statistical analysis

Cells were classified into phenotypic subsets, namely P21^+^/MCM2^−^ and CD105^+^/P21^+^/MCM2^−^, for analysis based on coexpression patterns. Each count was expressed as a percentage for individual patient samples by dividing it by the total population of cells from present on the slide. To determine the optimal threshold for stratifying patients based on P21+/MCM2− expression, we applied X-Tile (23), a bioinformatics tool designed for outcome-based biomarker assessment. This software analyses all possible cutoff points as a continuous data and selects the threshold that best separates patients into prognostically distinct groups, based on survival outcomes. Using time to relapse from the UK SORCE training cohort, X-Tile identified 2% as the most statistically significant cutoff point. This value was then used to categorize patients into low (<2%) and high (≥2%) P21+/MCM2− groups. The selected threshold was subsequently applied in the validation and test cohorts to ensure consistency and test prognostic reproducibility across independent datasets. Kaplan–Meier survival analysis of all the cohorts and two-sided *P* values were generated using R software (24). Hazard ratios (HR) and 95% confidence intervals (CI) were calculated using Cox proportional hazards regression analysis performed in SPSS (IBM SPSS Statistics, version 28.0.1.1).

## Results

### IHC and multiplex analysis of cell-cycle arrest in ccRCC

**Figure 1.**
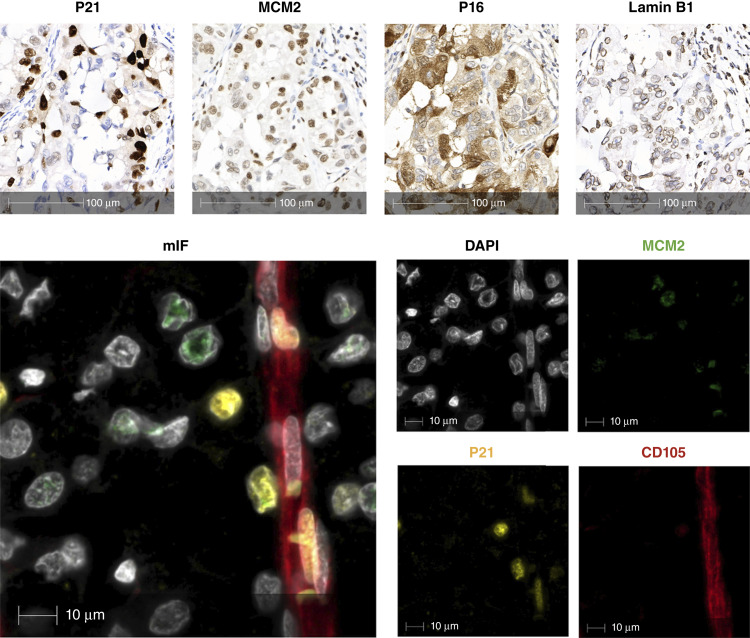
IHC detection of P21, MCM2, P16, and Lamin B1 (top) shows differential expression of cell-cycle arrest and proliferation markers in ccRCC tissue. mIF (bottom) reveals single-cell colocalization of P21 (yellow), MCM2 (green), and CD105 (red), with DAPI marking nuclei (gray).

IHC was performed on consecutive sections of ccRCC tissue obtained from the same patient. Established markers of cell-cycle regulation were used to distinguish between proliferative and nonproliferative states. As shown in Fig. 1, cells with strong nuclear expression of P21 and P16, key markers of cell-cycle arrest, were seen across the tumor section. By contrast, MCM2 was detected in a distinct subset of cells, consistent with ongoing cell-cycle activity. LAMIN B1 (*LMNB1*) exhibited heterogeneous nuclear positivity throughout the tumor, suggesting variability in nuclear membrane integrity and cell-cycle status across the section.

Within tumor regions, mIF revealed distinct cellular phenotypes based on combined P21 and MCM2 expression. Actively cycling cells were identified by MCM2 positivity without P21 (MCM2^+^/P21^−^), whereas noncycling cells expressed P21 but not MCM2 (P21^+^/MCM2^−^). A third, smaller subset coexpressed both markers (P21^+^/MCM^+^). CD105 was selected as a marker of activated endothelium (25). Nonetheless, its expression can extend beyond endothelial cells and may include subsets of tumor or stromal cells, potentially reflecting diverse microenvironmental roles (4). A subset of endoglin/CD105 labeled cells were P21^+^/MCM2^−^ (Fig. 1). In summary, both IHC and mIF revealed the coexistence of cycling and noncycling cell populations within ccRCC tissue, including nondividing CD105^+^ cells.

### P21^+^/MCM2^−^ cell percentage stratifies relapse risk in intermediate-risk ccRCC

To evaluate the prognostic relevance of cell cycle–arrested tumor cells in intermediate-risk ccRCC, as defined by the LS, we quantified the proportion of P21^+^/MCM2^−^ cells and assessed their association with time to relapse in two independent patient cohorts. The training cohort consisted of patients with intermediate-risk ccRCC SORCE cohort (*n* = 63), whereas the validation cohort comprised intermediate-risk patients with ccRCC from the Korean cohort (*n* = 71). We identified a 2% cutoff using X-Tile as the most effective discriminator of relapse risk in the SORCE training cohort. This threshold behaved as a binary classifier, effectively stratifying patients into high- and low-expressor groups.

**Figure 2.**
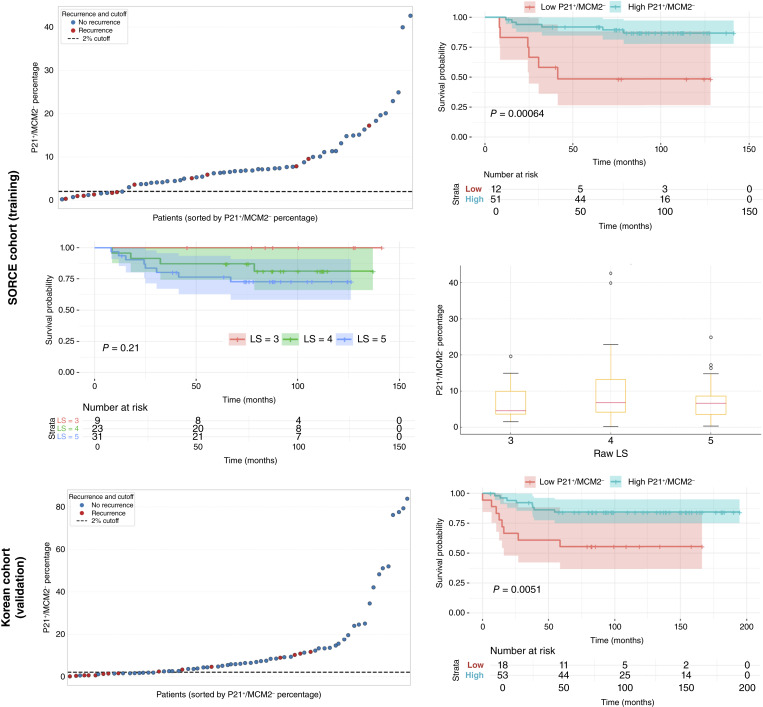
P21^+^/MCM2^−^ cell content improves risk stratification in intermediate-risk ccRCC. Kaplan–Meier survival analysis in the SORCE training cohort (*n* = 63) shows that patients with ≤2% P21^+^/MCM2^−^ cells experienced significantly higher relapse rates than those with >2% (*P* < 0.001) within 142 months. In contrast, stratification by the raw LS (LS 3–5) demonstrated a trend that was not statistically significant (*P* = 0.21). Box-and-whisker plots reveal overlapping distributions of P21^+^/MCM2^−^ cell percentages across LS subgroups. In the Korean validation cohort (*n* = 71), the same 2% threshold also significantly stratified outcomes (*P* = 0.005) within 195 months.

Figure 2 shows that in the SORCE training cohort, patients with >2% P21^+^/MCM2^−^ cell content demonstrated improved time to relapse (HR = 0.17; 95% CI, 0.06–0.54; *P* < 0.001), representing an 82.8% reduction in relapse risk compared with patients with low P21^+^/MCM2^−^ cell content. This difference was particularly pronounced within the first 5 years following surgery, during which only 8% of patients in the high-P21^+^/MCM2^−^ group experienced relapse compared with 50% in the low-expression group (*P* < 0.001). At 10 years, relapse remained substantially lower in the high-P21^+^/MCM2^−^ group (12%) compared with 50% in the low-expression group (*P* < 0.001).

To assess the prognostic performance of P21^+^/MCM2^−^ cell content relative to conventional clinical risk stratification, we compared time to relapse based on the LS and biomarker-defined subgroups within the intermediate-risk SORCE training cohort (Fig. 2). Kaplan–Meier analysis of patients stratified by raw LS values (LS 3, 4, and 5) demonstrated a trend toward increased relapse with higher scores. Also, Cox proportional hazards analysis showed that each 1-point increase in the raw LS showed no statistically significance, but there was a trend toward a 2.4-fold increase in relapse risk (HR = 2.40; 95% CI, 0.87–6.63; *P* = 0.21).

The association between P21^+^/MCM2^−^ and patient prognosis was independently validated in the Korean cohort, where the same 2% threshold effectively stratified patients into high- and low-risk groups (HR = 0.27; 95% CI, 0.10–0.72; *P* = 0.005). In this cohort, 15% of patients with high P21^+^/MCM2^−^ cell content relapsed within 5 years, compared with 44% in the low-risk group (*P* = 0.001). Together, these results demonstrate that a higher proportion of P21^+^/MCM2^−^ cells is associated with reduced relapse risk in intermediate-risk ccRCC.

Across both the SORCE training and Korean validation cohorts, most patients fell into the high-P21+/MCM2− group (∼75 to 81%), whereas a smaller subset (19%–25%), classified as low-expressors, exhibited significantly shorter time to relapse.

To evaluate the incremental prognostic value of the P21^+^/MCM2^−^ biomarker, we compared the discrimination of multiple prediction models incorporating elements of the LS with and without the biomarker. Across both the SORCE training cohort and the Korean validation cohort, addition of the biomarker consistently improved model discrimination. For example, the C-index of the LS increased from 0.64 to 0.73 in the SORCE cohort and from 0.73 to 0.78 in the Korean cohort when the biomarker was incorporated (Supplementary Table S1).

We evaluated whether CD105^+^/P21^+^/MCM2^−^ cells also carry prognostic value in ccRCC. As shown in Supplementary Fig. S1, using the same intermediate-risk LS training (SORCE) and validation (Korean) cohorts, we found a strong correlation between CD105^+^/P21^+^/MCM2^−^ and CD105^−^/P21^+^/MCM2^−^ cell percentages (r = 0.77 in SORCE, r = 0.99 in Korean). Stratification by the previously defined 2% threshold showed that patients with higher CD105^+^/P21^+^/MCM2^−^ levels had improved time to relapse in the intermediate-risk SORCE (HR = 0.13; 95% CI, 0.04–0.41; *P* < 0.001) and Korean (HR = 0.17; 95% CI, 0.06–0.48; *P* < 0.001) cohorts.

### P21^+^/MCM2^−^ tumor cells predict relapse across clear cell renal cell Leibovich risk categories

To determine whether the prognostic value of P21^+^/MCM2^−^ cell quantification extends beyond intermediate-risk disease, we validated our findings in two additional cohorts: a high-risk subgroup from the SORCE trial and the Scottish cohort (low-, intermediate-, and high-risk patients).

**Figure 3.**
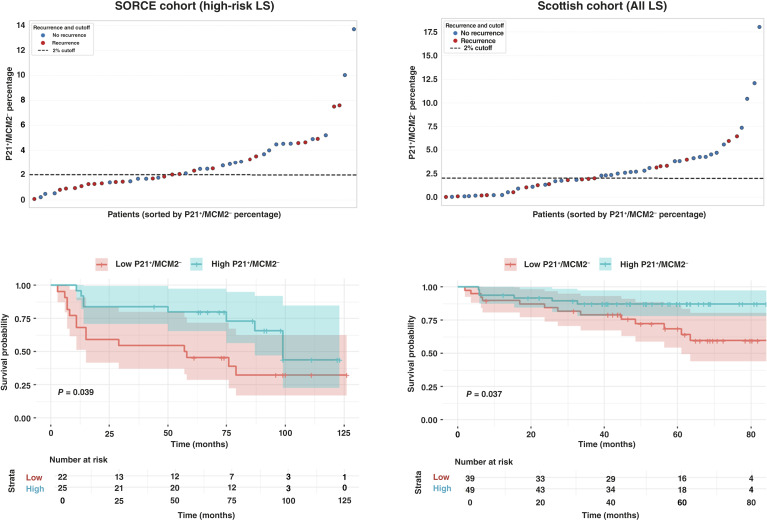
Validation of P21^+^/MCM2^−^ cell content as a prognostic biomarker across high-risk LS SORCE and pan-risk LS Scottish cohorts. In both high-risk patients and the pan-risk cohort, a 2% threshold stratifies relapse risk, with higher proportions associated with favorable survival outcomes.

Using the 2% threshold established for the training cohort, patients in the high-risk SORCE subgroup (*n* = 47) were stratified into low- and high-P21^+^/MCM2^−^ groups. Figure 3 shows that patients with >2% P21^+^/MCM2^−^ cells had significantly improved time to relapse compared with those with ≤2% (HR = 0.43; 95% CI, 0.19–0.99; *P* = 0.039). Within 5 years, 20% of high-P21^+^/MCM2^−^ patients relapsed versus 55% of those in the low-P21^+^/MCM2^−^ group (*P* = 0.023). At 10 years, relapse rates were 36% in the high-expression group and 64% in the low-expression group (*P* = 0.038).

In the Scottish cohort (*n* = 88), as shown in Fig. 3, the prognostic relevance of the 2% threshold was confirmed across all LS risk levels: patients with high P21^+^/MCM2^−^ cell content had significantly better outcomes than those with low levels (HR = 0.37; 95% CI, 0.14–0.98; *P* = 0.037). At the final 7-year follow-up time point, 12% of high-P21^+^/MCM2^−^ patients experienced relapse compared with 33% of those in the low-expression group.

The association of P21^+^/MCM2^−^ effect seen with CD105-positive cells was not seen when applied to ccRCC cases in the mixed intermediate- and high-risk Scottish cohort and SORCE high-risk group.

### Stratification by P21^+^/MCM2^−^ cells identifies differential responses to sorafenib adjuvant therapy in ccRCC

To examine whether P21^+^/MCM2^−^ cell content influences response to adjuvant therapy, we performed analysis on patients from the SORCE trial (*n* = 382) with intermediate- and high-risk ccRCC, as defined by the LS. Patients were stratified by a 2% threshold of P21^+^/MCM2^−^ cells and compared for time to relapse between those receiving sorafenib and those receiving placebo. The sorafenib group included patients who received either 1 year or 3 years of adjuvant treatment as part of the trial protocol.

**Figure 4.**
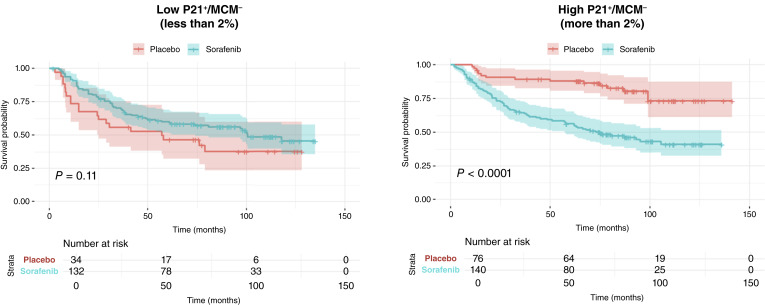
Stratification by P21^+^/MCM2^−^ status identifies differential responses to sorafenib in intermediate- and high-risk ccRCC. In patients with ≤2% P21^+^/MCM2^−^ cells, no significant benefit from sorafenib was observed (left). In contrast, patients with >2% of these cells had significantly worse outcomes when treated with sorafenib compared with placebo (right; *P* < 0.001).

As shown in Fig. 4, in patients with low P21^+^/MCM2^−^ levels (≤2%), there was no difference in time to relapse between treatment arms (HR = 1.51; 95% CI, 0.91–2.50; *P* = 0.11), indicating no benefit from sorafenib in this subgroup. However, in patients with high P21^+^/MCM2^−^ levels (>2%), those treated with placebo experienced more favorable time to relapse compared with those who received sorafenib (HR = 0.29; 95% CI, 0.16–0.50; *P* < 0.001), indicating that treatment with sorafenib removed the apparent benefit of having high P21^+^/MCM2^−^.

### Accumulation of nonproliferative P21^+^/MCM2^−^ cells in metastatic ccRCC

**Figure 5.**
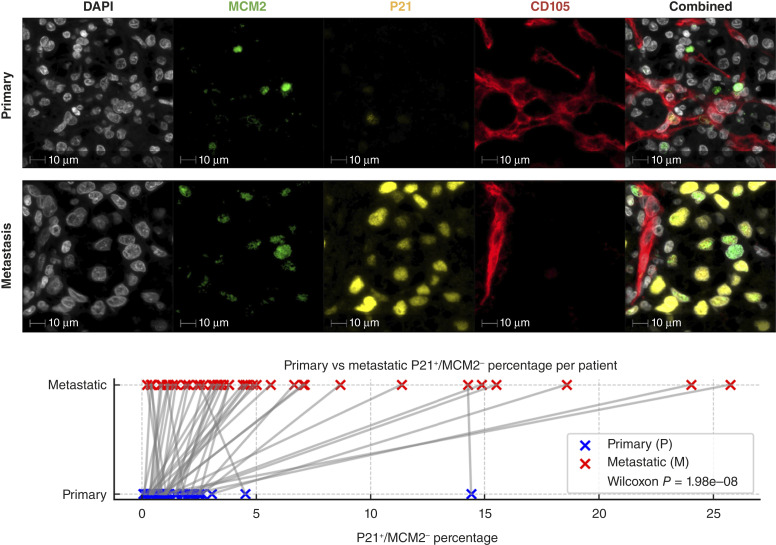
Higher P21^+^/MCM2^−^ cell percentage in metastatic ccRCC. Multiplex imaging shows greater P21^+^/MCM2^−^ expression in metastatic vs. primary tumors. In 35 of 41 patients, HALO AI–based quantification confirmed elevated percentages of P21^+^/MCM2^−^ at metastatic sites.

In a cohort of 41 cases, P21^+^/MCM2^−^ cells were infrequently detected in primary tumors, but in 35 of 41 patients (85%), the proportion of P21^+^/MCM2^−^ cells were higher in metastatic tissue than in the corresponding primary tumor (Wilcoxon signed-rank test statistic: 47, *P* < 0.001; Fig. 5).

## Discussion

This study describes a cellular phenotype in ccRCC that is defined by the presence of P21 and the absence of MCM2 (P21^+^/MCM2^−^). Although P21 is a well-established marker of G1 arrest in RCC (26), its combined assessment with MCM2, a proliferation licensing factor independent of p53 (27), provides a more refined stratification of tumor cell-cycle states. In patients treated only by surgery, a level higher than 2% P21^+^/MCM2^−^ is associated with better outcome; however, this benefit is lost if TKI treatment is introduced.

Within the intermediate-risk group of the SORCE cohort, stratification using the raw LS (LS 3–5) offered limited prognostic discrimination. Relapse risk remained poorly defined due to overlapping CIs and substantial heterogeneity within score subgroups. In contrast, P21^+^/MCM2^−^ cell content provided a clear biological distinction based on tumor cell state. Patients with low levels of P21^+^/MCM2^−^ cells exhibited relapse rates approaching 50% in both the SORCE and Korean cohorts—rates comparable with those seen in high-risk LS groups—yet these individuals would not have been identified using LS alone. This subgroup represents a population at elevated risk of relapse who may benefit from intensified surveillance, adjuvant therapy, and more precise stratification in clinical trials. These findings highlight that P21^+^/MCM2^−^ captures biologically relevant information that conventional clinicopathologic models overlook. Its ability to refine relapse risk within intermediate-risk patients and stratify outcomes across high-risk and all-risk ccRCC cohorts underscores its potential as a robust and clinically actionable biomarker.

In addition to cellular quiescence, this P21^+^/MCM2^−^ phenotype may also represent cellular senescence, a more persistent state of growth arrest frequently brought on by stresses such oxidative damage, oncogenic signaling, or treatment exposure (28). They can acquire a senescence-associated secretory phenotype (SASP), which is characterized by the release of growth factors, matrix-remodeling enzymes, and proinflammatory cytokines (29). By attracting immune cells to eradicate damaged or premalignant cells, SASPs may improve immune surveillance and encourage tumor suppression in specific situations (30). The more favorable results observed in patients with a higher percentage of P21^+^/MCM2^−^ cells in primary tumor may be explained by the possibility that these senescent-like cells produce a microenvironment that inhibits cancer growth or promotes immune-mediated clearance.

Furthermore, the identification of CD105^+^/P21^+^/MCM2^−^ cells, which showed a strong correlation with CD105^−^/P21^+^/MCM2^−^ cells and the same favorable prognostic association, suggests that this phenotype is not confined to the epithelial tumor compartment. Given the well-established use of CD105 as a marker of activated endothelium (31), it is likely that these cells are localized within the vascular or stromal components of the tumor microenvironment. This raises the possibility that cell-cycle arrest, and potentially senescence, may occur in nonepithelial compartments such as the endothelium, where it could influence angiogenic balance, immune cell infiltration, and tumor progression (32).

We have shown that presence of P21^+^/MCM2^−^ cells in the resected primary tumor confers a better prognosis but that the effect is lost after treatment with adjuvant TKI. As the nephrectomy in every case was undertaken with curative intent, the explanation for why P21 and MCM2 are biomarkers must indicate a relationship between what is measured in the primary tumor and what is present in the micrometastases that are not clinically apparent. In 85% of the paired primary/metastatic samples (which are clinically apparent by definition, not micrometastases), the frequency of P21^+^/MCM2^−^ cells were higher in the metastatic tissue. Sorafenib has been shown to suppress P21 expression in RCC cell lines (33) and to selectively target senescent cells (34), suggesting that it may disrupt prodormancy signaling mediated by SASP factors or interfere with local immune surveillance. In patients with high P21^+^/MCM2^−^ content, TKI therapy may lead to the unintended elimination of regulatory or suppressive cell populations, allowing micrometastases to clinically declare themselves. Although adjuvant TKIs are no longer considered standard of care for ccRCC, the finding that TKIs may adversely affect outcomes in patients whose tumors exhibit a phenotypic feature present in as little as 2% of cells underscores the nuanced role of the tumor microenvironment in modulating therapeutic response. This raises the possibility that similar microenvironmental dynamics may influence treatment efficacy and resistance in the metastatic setting, warranting further investigation.

In conclusion, our study positions P21^+^/MCM2^−^ cell content as a possible biomarker that may reflect the dynamic interplay between proliferation arrest, senescence, and therapy response in ccRCC. The integration of this marker into clinical practice, as supported by improved model discrimination in C-index analyses, could support more refined prognostic models and avoid ineffective or potentially harmful treatments.

## Supplementary Material

Supplementary Figure 1 A P21^+^/MCM2^−^ Subpopulation of CD105^+^ Cells Predicts Favourable Outcome in Renal Cell Carcinoma

Supplementary Table 1 Comparison of prognostic model discrimination with and without the P21^+^/MCM2^−^ biomarker

## Data Availability

The data generated in this study are publicly available in the BioImage Archive. The SORCE cohort is available under accession S-BIAD2453 (DOI: 10.6019/S-BIAD2453); the Korean ccRCC cohort under S-BIAD2452 (DOI: 10.6019/S-BIAD2452); the Scottish ccRCC cohort under S-BIAD2454 (DOI: 10.6019/S-BIAD2454); and the metastatic cohort under S-BIAD2455 (DOI: 10.6019/S-BIAD2455). All datasets are released under a CC0 license.
