# Supplementary material for: Multiplexed P21/MCM-2 Detection Predicts Relapse and May Identify Tyrosine Kinase Inhibitor–Resistant Patients in Clear Cell Renal Cell Carcinoma
Source: Cancer Res Commun. 2026 May 7;6(5):1061–70. doi: 10.1158/2767-9764.CRC-25-0805 (PMC13150819; doi:10.1158/2767-9764.CRC-25-0805)
Supplement: Supplementary Figure 1 — A P21+/MCM2− Subpopulation of CD105+ Cells Predicts Favourable Outcome in Renal Cell Carcinoma [file crc-25-0805_supplementary_figure_1_suppsf1.docx]

## A P21⁺/MCM2⁻ Subpopulation of CD105⁺ Cells Predicts Favourable Outcome in Renal Cell Carcinoma

We investigated whether the population of CD105⁺/P21⁺/MCM2⁻ cells could reflect overall tumour cell cycle arrest and carry prognostic value in clear cell renal cell carcinoma (ccRCC). The same Intermediate risk Leibovich Risk Score (LS) training and validation cohorts were used to investigate the prognostic value of identifying and quantifying CD105⁺/P21⁺/MCM2⁻ cells. There was a strong correlation between the percentage of CD105⁺/P21⁺/MCM2⁻ cells and CD105⁻/P21⁺/MCM2⁻ cells across patients. As shown in figure 6, In the training SORCE cohort, the Pearson correlation coefficient was r = 0.77, and in the validation Korean cohort, this correlation was r = 0.99, indicating highly consistent alignment between these subsets.

Patients were stratified into high and low CD105⁺/P21⁺/MCM2⁻ groups based on the 2% threshold previously defined for prognostic discrimination. Kaplan–Meier survival analysis demonstrated that individuals with higher levels of these cells experienced significantly improved recurrence-free survival in both the UK arm of SORCE (n = 63, p < 0.0001) and Korean (n = 71, p = 0.0001) cohorts. In the SORCE cohort, only 8% of patients in the high CD105⁺/P21⁺/MCM2⁻ group recurred within 5 years, compared to 50% in the low group (p = 0.00004). At 10 years, recurrence remained significantly lower in the high group (10%) compared to 58% in the low group (p = 0.0002). Similarly, in the Korean cohort, 12% of patients with high CD105⁺/P21⁺/MCM2⁻ content recurred within 5 and 10 years, whereas 56% of those in the low group experienced recurrence (p = 0.0007). Together, these findings indicate that CD105⁺/P21⁺/MCM2⁻ cells may reflect a non-proliferative, vascular phenotype associated with durable suppression of disease recurrence in ccRCC.


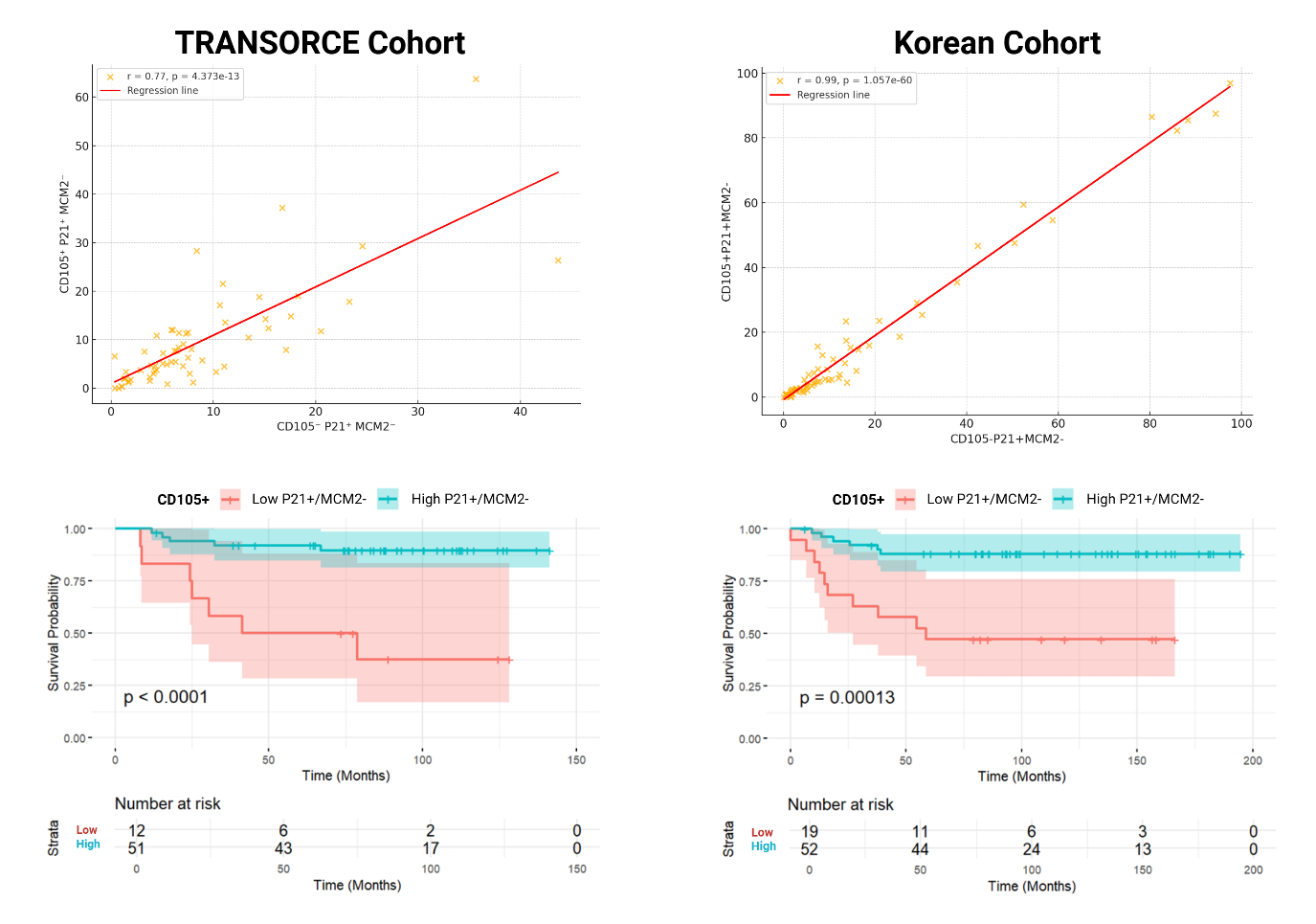


**Supplementary figure 1**. Prognostic value of CD105⁺/P21⁺/MCM2⁻ cells in intermediate risk LS clear cell RCC. In both intermediate-risk Leibovich score (LS) ccRCC cohorts—SORCE (training) and Korean (validation)—CD105⁺/P21⁺/MCM2⁻ cell content demonstrated a strong correlation with overall tumour P21⁺/MCM2⁻ burden. Patients with >2% of this subset consistently showed improved recurrence-free survival, supporting its prognostic significance.
