# Supplementary material for: Multiplexed P21/MCM-2 Detection Predicts Relapse and May Identify Tyrosine Kinase Inhibitor–Resistant Patients in Clear Cell Renal Cell Carcinoma
Source: Cancer Res Commun. 2026 May 7;6(5):1061–70. doi: 10.1158/2767-9764.CRC-25-0805 (PMC13150819; doi:10.1158/2767-9764.CRC-25-0805)
Supplement: Supplementary Table 1 — Comparison of prognostic model discrimination with and without the P21+/MCM2− biomarker [file crc-25-0805_supplementary_table_1_suppst1.docx]

## Comparison of prognostic model discrimination with and without the P21⁺/MCM2⁻ biomarker

We evaluated the prognostic performance of multiple Cox proportional hazards models for disease-free survival in the SORCE training cohort and the Korean validation cohort. Models included individual clinicopathological variables and combinations of elements of the Leibovich score (pathological stage, ISUP grade, tumour size, and necrosis), assessed both with and without inclusion of the P21⁺/MCM2⁻ biomarker. Model discrimination was quantified using Harrell’s C-index to determine the incremental prognostic value of the biomarker beyond established clinicopathological factors.

| **Variables** | **SORCE C-index** | **Korean C-index** |
| --- | --- | --- |
| P21⁺/MCM2⁻ | **0.71** | **0.65** |
| Leibovich | 0.64 | **0.73** |
| Leibovich + P21⁺/MCM2⁻ | **0.73** | **0.78** |
| T stage | 0.66 | 0.69 |
| Stage + P21⁺/MCM2⁻ | **0.75** | **0.76** |
| Stage + ISUP grade | 0.62 | 0.73 |
| Stage + grade + P21⁺/MCM2⁻ | **0.77** | **0.77** |
| Stage + grade + size | 0.63 | **0.77** |
| Stage + grade + size + P21⁺/MCM2⁻ | **0.77** | **0.79** |

**Supplementary Table 1.** Harrell’s C-index is reported for each Cox proportional hazards model as a measure of discrimination for disease-free survival, with higher values indicating improved predictive accuracy. Models include individual clinicopathological variables and combinations of elements of the Leibovich score (pathological stage, ISUP grade, tumour size, and tumour necrosis), evaluated with and without inclusion of the P21⁺/MCM2⁻ biomarker (defined using a >2% threshold). Results are shown for both the SORCE training cohort and the Korean validation cohort to assess the incremental prognostic value of the biomarker across independent datasets.
